# Exploring depression in Alzheimer’s disease: an Italian Delphi Consensus on phenomenology, diagnosis, and management

**DOI:** 10.1007/s10072-023-06891-w

**Published:** 2023-07-04

**Authors:** Alessandro Padovani, Angelo Antonini, Paolo Barone, Giuseppe Bellelli, Andrea Fagiolini, Luigi Ferini Strambi, Sandro Sorbi, Fabrizio Stocchi

**Affiliations:** 1 Neurology Unit, Department of Clinical and Experimental Sciences, University of Brescia, DSCS-c/o U.O. Neurologia 2, Spedali Civili 1, 25121 Brescia, Italy; 2 Parkinson and Movement Disorders Unit, Center for Neurodegenerative Diseases (CENSE), Department of Neuroscience, University of Padua, Padua, Italy; 3 Center for Neurodegenerative Diseases (CEMAND), Neuroscience Section, University of Salerno, Salerno, Italy; 4 School of Medicine and Surgery, University of Milano-Bicocca, 20126 Milan, Italy; 5 Acute Geriatric Unit, IRCCS San Gerardo, 20900 Monza, Italy; 6 Department of Molecular and Developmental Medicine, University of Siena School of Medicine, Siena, Italy; 7 IRCCS San Raffaele Scientific Institute, Department of Clinical Neurosciences, Neurology-Sleep Disorders Centre, Milan, Italy; 8 “Vita-Salute” San Raffaele University, Milan, Italy; 9 Department of Neuroscience, Psychology, Drug Research and Child Health, University of Florence, Azienda Ospedaliero-Universitaria Careggi, Largo Brambilla, 3, 50134 Florence, Italy; 10 IRCCS Fondazione Don Carlo Gnocchi, Florence, Italy; 11 University and Institute for Research and Medical Care, IRCCS San Raffaele, Rome, Italy

**Keywords:** Alzheimer’s disease, Depression, Antidepressant therapy

## Abstract

**Background:**

In Alzheimer’s disease (AD), the progressive cognitive impairment is often combined with a variety of neuropsychiatric symptoms, firstly depression. Nevertheless, its diagnosis and management is difficult, since specific diagnostic criteria and guidelines for treatment are still lacking. The aim of this Delphi study is to reach a shared point of view among different Italian specialists on depression in AD.

**Methods:**

An online Delphi survey with 30 questions regarding epidemiology, diagnosis, clinical features, and treatment of depression in AD was administered anonymously to a panel of 53 expert clinicians.

**Results:**

Consensus was achieved in most cases (86%). In the 80% of statements, a positive consensus was reached, while in 6% a negative consensus was achieved. No consensus was obtained in 14%. Among the most relevant findings, the link between depression and AD is believed to be strong and concerns etiopathogenesis and phenomenology. Further, depression in AD seems to have specific features compared to major depressive disorder (MDD). Regarding diagnosis, the DSM 5 diagnostic criteria for MDD seems to be not able to detect the specific aspects of depression in AD. Concerning treatment, antidepressant drugs are generally considered the main option for depression in dementia, according to previous guidelines. In order to limit side effects, multimodal and SSRI antidepressant are preferred by clinicians. In particular, the procognitive effect of vortioxetine seems to be appealing for the treatment of depression in AD.

**Conclusions:**

This study highlights some crucial aspects of depression in AD, but more investigations and specific recommendations are needed.

## Introduction

Dementia currently affects 24 million individuals worldwide, and, with population aging, its prevalence is expected to quadruple by 2050 [1]. Alzheimer’s disease (AD) is the leading cause of dementia in older adults, while other types such as vascular, Lewy body, and frontotemporal dementia (FTD) are less common. While progressive cognitive impairment is the hallmark of AD, neuropsychiatric symptoms affect almost all patients and are persistent [2]. Depression is one of the most common neuropsychiatric symptoms in AD, associated with institutionalization and mortality [3].

A recent meta-analysis established that the prevalence of major depression was 15.9% and 14.8% in all-cause dementia and in AD, respectively [4]. Multiple lines of evidences suggest that the link between depression and neurodegenerative disease is strong. It is well known that the presence of chronic depression increases the risk of dementia later in life [5], while the emergence of new depressive symptoms in older adults can precede the diagnosis of cognitive decline and dementia. Further, the occurrence of major depressive disorder (MDD) in AD may accelerate cognitive and functional decline [6]. Finally, depression has a strong negative influence on conversion to AD in patients with mild cognitive impairment [7]. Despite these data, depression in AD is still underdiagnosed and, therefore, undertreated most likely as a consequence of the lack of consistent diagnostic criteria to assess depression in this context [8]. A major limitation to the diagnosis of depression in AD is the potential overlap between symptoms of depression and symptoms resulting from the cognitive and functional decline. Another limitation could be the paucity of instruments specifically designed to assess depression in dementia.

Once depression has been diagnosed in AD patients, the treatments are still controversial. Antidepressants represent the first choice of treatment for AD patients with depression. This is due to a lack of alternative treatment options and a perception that antidepressants are effective in this population [9]. On the other hand, there is not clear evidence from systematic reviews and meta-analyses to support this practice. A recent meta-analysis including 7 studies found no statistically significant difference between antidepressants and placebo [10]. At the same time, clear indications for preferring a specific antidepressant drug group over another are still lacking and antidepressant drug is chosen on the basis of clinical practice guidelines (CPG) or consensus on either AD or depression, or on the basis of the empirical expertise of clinicians [11]. However, the most recent multimodal antidepressant vortioxetine was not included in previous meta-analysis [10] but it may be considered as a promising alternative for the treatment of depression in patients with AD. In fact, in addition to blocking the serotonin transporter (SERT), vortioxetine has a complex interaction with different 5-HT receptors leading to the modulation of different neurotransmitters, including acetylcholine (Ach) [12]. Because of these pharmacodynamics characteristics, a possible role on improving cognitive deficits has been described and investigated. Some previous studies demonstrated the efficacy of vortioxetine on cognitive symptoms in elderly depressed patients [13–16]. Two studies investigated the efficacy on cognitive functions in patients with mild or moderate AD, with conflicting results [17, 18]. Finally, the role of antidementia medications (acetylcholinesterase inhibitors) on depressive symptoms is still debated [19, 20]. In summary the approach to depression in AD is quite different among physicians and clear guidelines are still lacking. Consequently this topic has been the objects of different Consensus Delphi in many countries [21] in different settings [22].

The aim of this Delphi study is to reach a shared point of view among different specialists on the diagnosis and management of depression in AD in Italy.

## Methods

The Delphi method is an approach frequently used in scientific and medical contexts aimed at reaching consensus among a group of experts, when scientific evidence is limited or conflicting [23, 24]. Our study is a modified Delphi study with only one round, therefore different from the classic model which provides a series of “*n*” rounds to allow respondents to review their initial assessments until a certain degree of convergence of the panel’s views. The aim was to measure, within the Italian reality, the level of consensus/perception on some aspects of the management of depression in Alzheimer’s disease. The study consisted of the administration of an online survey to a panel of specialists in neurology, psychiatry, and geriatrics. A steering committee composed by nine distinguished physicians (7 neurologist, 1 psychiatrist, 1 geriatrician) in the field of Alzheimer’s disease formulated 30 questions. The questions were discussed during an online meeting, reflecting the more debated aspects of phenomenology, diagnosis, and management of depression in AD in order to encourage a food for thought among panelists. The questionnaire was administered anonymously and the 30 statements were grouped in 4 sections (Depression and neurodegenerative disease—Clinical features of depression in AD—Diagnostic criteria of depression in AD—Treatment of depression in AD). For each statement of the questionnaire, participants had to express their degree of agreement or disagreement according to the following 5-point Likert scale: 1 = strongly disagree; 2 = disagree; 3 = agree; 4 = more than agree; 5 = strongly agree. In accordance with the Delphi standards, a positive consensus were reached when the sum of items 3, 4, and 5 (agree) reaches 66%, while a negative consensus were obtained if more than 66% of answers were 1 ad 2 (disagree). No consensus was reached when the sum of the responses for a negative consensus (1 and 2) or a positive consensus (3, 4, and 5) was <66%. Participants responded to the 30 items of the questionnaire on only one round. The second round was not administered because there is no available literature on the 4 items that have not reached the consensus.

## Populations

Nine distinguished physicians (7 neurologist, 1 psychiatrist, 1 geriatrician) in the field of Alzheimer’s disease constituted the Steering Committee that designed the study, drafted the questionnaire, analyzed, and discussed the results. Further, the Steering Committee identified the panel of specialists that participate to the Delphi method with the following skills: neurologist or geriatrician; at least 10-year experience on Alzheimer’s disease—visiting at least 10 patients per week; gender balance (50% women)—equally allocated in the various Italian areas—selected throughout the study group on the base of academic roles, scientific publications. Each member of the Steering Committee proposed 5 to 8 physicians with these skills as panelists.

Fifty-three physicians (41 neurologist, 12 geriatrist) were invited by email to answer to the online Delphi questionnaire. No personal data were collected from the panel of experts, with the exception of the email addresses that were used only for sending invitation and reminder emails. All the data from the questionnaire were analyzed in an anonymous way.

## Results

The Delphi survey was presented to the panel of experts. Of 53 invited physicians, 46 completed the questionnaire. Data from the Delphi questionnaire are reported: overall, consensus was achieved in most cases (26 statements; 86%). Specifically, 24 statements (80%) obtained positive consensus, while only 2 statements (6%) achieved a negative consensus. Finally, no consensus was obtained in 4 statements (14 %). Tables 1, 2, 3, and 4 show all Delphi statements according to 4 sections (Depression and neurodegenerative disease—Clinical features of depression in AD—Diagnostic criteria of depression in AD—Treatment of depression in AD). No consensus or consensus, and if so, either agreement or disagreement is reported for each statement. The distribution of preferences among the five scores is also specified.

**Table 1:** Depression and neurodegenerative disease

	1	2	3	4	5
*(1.1) In the elderly, depression can precede the onset of a neurodegenerative disease.*	0%		100%		
	0	0	5	13	28
*(1.2) Patients affected by neurodegenerative disease (as Alzheimer’s disease) often have depressive symptoms.*	4%		96%		
	0	2	7	14	23
*(1.3) Depressive symptoms are directly related to the neuropathological features of Alzheimer’s disease.*	24%		76%		
	1	10	18	13	4
*(1.4) Depressive symptoms are an integral part of the symptomatology of Alzheimer’s disease.*	17%		83%		
	2	6	18	11	9
*(1.5) In Alzheimer’s disease, depressive symptoms meet the diagnostic criteria of depressive disorder as described by DSM-V.*	61%		39%		
	2	26	12	5	1

**Table 2:** Clinical features of depression in AD

	1	2	3	4	5
*(2.1) Depressive symptoms and anxiety can be due to cholinergic deficiency.*	33%		67%		
	3	12	20	10	1
*(2.2) In patients with AD depressive symptoms and anxiety can make difficult a suitable assessment of cognitive disturbances.*	0%		100%		
	0	0	9	25	12
*(2.3) Patients with medical history of depressive disorder or bipolar disorder have an increased risk of developing AD.*	22%		78%		
	1	9	14	13	9
*(2.4) In patients with AD, depression is always associated to executive deficits.*	37%		63%		
	3	14	1	12	0
*(2.5) In patients with AD, depressive symptoms and the extent of cognitive impairment are not correlated.*	26%		74%		
	2	10	17	12	2
*(2.6) Suicidal ideation is not typical in AD.*	20%		80%		
	0	9	9	20	8
*(2.7) In patients with AD, anhedonia is the most typical symptom of depression.*	33%		67%		
	2	13	16	9	6
*2.8 The risk of developing a major depressive disorder is the same in patients with AD and in general population.*	72%		28%		
	10	23	10	2	1

*AD* Alzheimer’s disease

**Table 3:** Diagnostic criteria of depression in AD

	1	2	3	4	5
*(3.1) In patients with AD, the assessment of specific diagnostic scales (as GDS; Beck, PHQ9) is useful to identify depressive symptoms.*	13%		87%		
	1	5	14	15	11
*(3.2) It is important to recognize cognitive disturbances associated to depressive symptoms.*	0%		100%		
	0	0	3	15	28
*(3.3) In AD, a depressive disorder is diagnosed just according to clinical examination.*	50%		50%		
	5	18	13	7	3

*AD* Alzheimer’s disease

**Table 4:** Treatment of depression in AD

	1	2	3	4	5
*(4.1) In patients with Alzheimer’s disease, the therapeutic approach to depressive symptoms provides for the optimization of ongoing therapy as a first line.*	15%		85%		
	1	6	11	14	14
*(4.2) In AD, age of patients conditions the employment of tricyclic antidepressant drugs at therapeutic doses.*	41%		59%		
	11	8	9	13	5
*(4.3) In patients with AD, SSRI and SNRI can worsen cognitive and motor symptoms.*	70%		30%		
	9	23	9	5	0
*(4.4) According to their specific efficacy and tolerability in AD, SSRI and multimodal antidepressant drugs are a valid therapeutic choice for the treatment of depression.*	2%		98%		
	0	1	13	22	10
*(4.5) In patients with AD, some clinical features as a proved efficacy on anhedonia should be a selection criteria for the treatment of depression.*	15%		85%		
	0	7	18	15	6
*(4.6) In patients with AD, some clinical features as a proved efficacy on cognitive symptoms should be a selection criteria for the treatment of depression.*	2%		98%		
	0	1	10	18	17
*(4.7) In patients with AD, the tolerability and safety profile of antidepressant drug (as weight, sleep, genito-urinary functions) should be considered a significant question.*	0%		100%		
	0	0	6	18	22
*(4.8) In patients with AD, the treatment of depression may last 3 months at least.*	9%		91%		
	10	23	10	2	1
*(4.9) Therapy with SSRI can cause a restless leg syndrome and/or can cause a sleep fragmentation by increasing periodic limbs movements.*	30%		70%		
	2	12	16	8	8
*(4.10) Cholinergic and dopaminergic drugs are effective for the treatment of depressive symptoms in AD.*	33%		67%		
	3	12	16	14	1
*(4.11) In patients with AD, cognitive-behaviour therapy is effective to treat depressive symptoms.*	30%		70%		
	5	9	23	9	0
*(4.12) The association of cholinesterase inhibitors and serotoninergic antidepressant drugs could have a synergistic effect on the treatment of depression in patients with AD.*	2%		98%		
	0	1	19	19	7
*(4.13) In patients with AD, vortioxetine is a multimodal antidepressant drug that could be effective on mood but also on anhedonia.*	4%		96%		
	0	2	14	20	10
*(4.14) In patients with AD, vortioxetine is a multimodal antidepressant drug that could be effective on cognitive symptoms.*	7%		93%		
	1	2	15	20	8

*AD* Alzheimer’s disease, *SSRI* selective serotonin reuptake inhibitors

In the section “Depression and neurodegenerative disease,” participants achieved an agreement in 4 statements (80%), while 1 statement reached no consensus (20%) (Table 1). Regarding the section “Clinical features of depression in AD,” 6 statements (75%) achieved an agreement, 1 (12.5%) a disagreement, and 1 resulted in no consensus (12.5%) (Table 2). In the field of Diagnostic criteria of depression in AD (Table 3), 2 agreements (66.6%) and 1 no consensus (33.3%) was reported. Most participants agreed on the importance of specific diagnostic tools, while it was no consensus if the clinical judgment alone is enough to diagnose depression. Finally, regarding treatments, an agreement was obtained in 12 statements (86%), a disagreement in 1 statement (7%), and a no consensus in 1 statement (7%) (Table 4).

## Discussion

The aim of this Delphi study was to reach a common point of view on depression in AD among a large numbers of specialists and to encourage a serious consideration on the most debated questions that make difficult the management of a depressed patient with AD. Globally considered, the results of the present study are in the expected direction, reflecting the current knowledge and the literature on depression in AD. Hereafter, the results are discussed according to the principal items.

### Depression and neurodegenerative disease

Even if the issue of whether depression is an AD-associated prodromal condition or a risk factor is controversial [4], all the participants agreed on the presence of a temporal link between depression and neurodegenerative disease (s.1.1). This result is in line with a growing body of evidence and with the positioning of medical societies such as the European Federation of Neurological Societies (EFNS), the National Institute for Health and Care Excellence (NICE), the American Psychiatric Association (APA), and the World Federation of Societies of Biological Psychiatry (WFSBP).

These medical societies recommend regular depressive symptom appraisal in older adults with dementia and assessment of other secondary causes. Most specialists also believed that depressive symptoms are directly related to the neuropathological features of Alzheimer’s disease (s.1.3). Nevertheless, previous studies investigating the link between depressive symptoms and neuropathological changes in clinical AD found conflicting results [25, 26]. Some prospective-cohort studies described that patients with AD and history or diagnosis of depression had higher levels of neuritic plaques and neurofibrillary tangles [25]; instead other cross-sectional studies failed to associate CSF biomarkers of AD and depressive symptom in AD [26]. Regardless of the association between AD pathology and depression, depressive symptoms are recognized as an integral part of the symptomatology of AD that frequently affects patients (s.1.2, s.1.4); therefore, most specialists believed that AD does not affect only cognitive dimension. After all, since 1996 the spectrum of non-cognitive and non-neurological symptoms of dementia, such as agitation, aggression, psychosis, depression, and apathy, was described with the term “Behavioural and Psychological Symptoms in Dementia” (BPSD) by the International Psychogeriatric Association (IPA) [27].

The most debated question of this section concerns the use of the diagnostic criteria of major depressive disorder (MDD) as described by DSM-V to diagnose depression in AD (s.1.5). No consensus was reached among the specialists, as well as a clear recommendation from scientific reports is still lacking. In fact, these criteria appear not specific in the context of AD, since they do not take in account the presence of the cognitive impairment and the overlap between cognitive and affective symptoms. Consequently, some peculiar features of depression in AD are not considered: in example, suicidal ideation and sense of guilt are infrequent, while anhedonia is often exacerbated [8]. A workgroup convened by the National Institutes of Mental Health (NIMH) proposed standardized diagnostic criteria for depression in AD (NIMH-dAD) [28]. These are similar to the DSM-V criteria for major depression, but with the inclusion of irritability and social isolation replacing loss of libido, and with loss of pleasure in response to social contact replacing loss of interest. Nevertheless, the NIMH-dAD criteria are frequently unknown and are not regularly used by specialists.

### Clinical features of depression in AD

In the section “Clinical features of depression in AD,” the opinion of the specialists on the phenomenology of depression in AD is explored. Even if most of statements reached a consensus, the major number of preferences belongs to the point 3 (agree) of the 5-point Likert scale, suggesting a not very strong consensus on these themes.

The affective dimension of depression in AD is well defined in the opinion of specialists that identified the presence of anhedonia and the absence of suicidal ideation as remarkable features of depression in AD (statement 2.6, 2.7), according to the phenomenology of depression in AD as described by many authors [8].

The link between cognitive and affective symptoms is more debated. According to the Cumming’s hypothesis, affective symptoms of AD are ascribed to the cholinergic deficiency (s.2.1) [29].

Therefore, depression is considered as a distinct entity from cognitive impairment, such that depressive symptoms and the extent of cognitive impairment are not correlated in the opinion of the participants (s.2.5). Effectively, a systematic review did not find a significant association between severity of AD and prevalence of depressive symptoms in three of the four high quality included studies [30]. Even if frontal and prefrontal executive deficit is significantly impaired during, and between, depression episodes in not demented individuals with major depressive disorder, nevertheless no consensus was reached on the presence of executive deficits in depressed patients with AD (s.2.4). Probably the participants believed that it is not possible to distinguish executive deficits due to AD from that due to depression. This data reveals a more general limitation of clinical and neuropsychological assessment of depressed patients with AD that concerns the complex overlap between cognitive and affective dimension. As confirmation, most specialists believed that in patients with AD, depressive symptoms and anxiety can make difficult a suitable assessment of cognitive disturbances (s.2.2), underlying the challenge to separate cognitive and affective aspects and the complex interaction between the two. Actually different profiles between cognitive deficits in depression and dementia have been frequently described [31], but most specialists feel not confident in using these investigations in clinical practice. Finally, the bidirectional relationship between AD and major depressive disorder is investigated. In this context, answers of participants were in the expected direction. On one side, participants agreed on the increased risk of developing AD in patients with medical history of depressive disorder or bipolar disorder (s.2.3); on the other they believed that the risk of developing a major depressive disorder is not the same in patients with AD and in general population (s.2.8). Concerning the first statement, it is in line with most studies that demonstrate that a history of both depression and bipolar disorder is associated with significantly higher risk of dementia in older adults [4]. Further, when depression occurs late in life, it acts as a dementia risk factor (cause) or an early sign (prodrome) of an underlying neurodegenerative disease typically associated with AD. This interconnection seems to be dependent on age, depression severity, and success of antidepressant treatment [32]. It has been considered that late-onset depression might be a prodromal condition of AD due to short time lag between the onsets. A recent meta-analysis, which included 23 community-based prospective cohort studies, concluded that late-life depression significantly increased the risk of AD incidence for 1.65 fold, even after considering all possible confounders [33]. On the other hand, if there was a long time lag between the onsets, depression was usually considered more of a risk factor than a prodromal condition [4]. On the other hand, the participants appear to be aware of the high prevalence of depression in patients with AD (s.2.8), according to a recent meta-analysis [3] that indicates that individuals with dementia have an increased prevalence of major depressive disorder (14.8%) when compared to individuals without dementia, where depression prevalence is approximately 1.8% [34]. Further, the prevalence of depression in AD varies greatly in different investigations, depending on the diversity in research methodology, diagnostic criteria for depression, and patient samples. For example, studies using specific diagnostic criteria for depression in AD such as NIMH-dAD criteria [28] suggested higher depressive prevalence of around 50% [35], while those using categorical diagnostic criteria such as the Diagnostic and Statistical Manual of Mental Disorders (DSM-V) and the International Classification of Diseases (ICD-10) indicated comparatively lower prevalence of around 10–40% [36].

### Diagnostic criteria of depression in AD

All the experts agreed on the importance of recognizing the cognitive disturbances associated to depressive symptoms (s.3.2). Patients with major depressive disorder often experience impairment in cognitive functions in several domains, including executive functioning, processing speed, concentration/attention, learning, and memory [37], such that impairment in cognition is included in the diagnostic criterion for major depressive disorder in the Diagnostic and Statistical Manual 5. Nevertheless, low cognitive performance in depressed adults can be an artifact for AD diagnosis. On the other hand, the absence of cognitive improvement upon depressive symptom remission may suggest an ongoing neurodegenerative process; considering that around one third of the adult population with depression is diagnosed with concomitant mild cognitive impairment (MCI) [38], the recognition of cognitive impairment in depressed patients is crucial to drive the follow-up of these patients.

The most debated question is how depressive symptoms have to be diagnosed in patients with AD. In fact, no consensus was reached regarding the use of only clinical examination as diagnostic tool, while most specialists agreed on the utility of specific diagnostic scales. As already discussed, these results confirmed that the phenomenology of depressive symptoms in AD runs away a clear systematization and useful diagnostic tool and diagnostic criteria are still lacking. Consequently, the diagnostic scales as the Cornell Scale for Depression in Dementia, the Geriatric Depression Scale, or the Beck Depression Inventory, can be useful, but clinicians are still confident in their clinical judgment, underlying the difficulty to put depressive symptoms in the context of the cognitive impairment of AD.

### Treatment of depression in AD

In this section, the opinion of specialists on different therapeutic alternatives for the treatment of depression in AD was explored. All the participants agreed that the choice of the antidepressant drug for a depressed patient with AD must be driven by its tolerability and safety profile (s.4.7). In fact, a growing body of evidence reveals that the use of antidepressant drugs is associated with an increased risk of adverse effects and events in older people, such as nausea, diarrhea, falls, hip fractures, cardiovascular events, all-cause mortality, and orthostatic hypotension [39]. Quite the opposite, the efficacy of antidepressant drugs in AD has not been proven by a recent review [40]. According to these data, the guidelines of the APA Work Group on Alzheimer’s Disease and other Dementias for treating depression in AD suggest the use of antidepressants with minimal and the least severe adverse effects as the first-line approaches [41]. Regarding the side effects of antidepressant drugs, there is a mild consensus on the association between use of selective serotonin reuptake inhibitors (SSRI) and restless leg syndrome (s.4.9). A recent study found a potential harmful association between movement disorders and use of various antidepressants (mirtazapine, vortioxetine, amoxapine, phenelzine, tryptophan, fluvoxamine, citalopram, paroxetine, duloxetine, bupropion, clomipramine, escitalopram, fluoxetine, mianserin, sertraline, venlafaxine, and vilazodone) [42]. Nevertheless, the specialists do not believe that SSRI and serotonin-norepinephrine reuptake inhibitor (SNRI) such as multimodal one can worsen cognitive and motor symptoms in AD (s.4.3). Maybe this inconsistency is due to the belief that cognitive symptoms can take advantage from antidepressant therapy, as demonstrated by some previous studies [12]. Further, the increased occurrence of REM behavior disorder during antidepressant therapy can make difficult the differential diagnosis between Alzheimer’s disease and dementia with Lewy bodies. No consensus was reached in the field of tricyclic antidepressant. It is not clear, in fact, if specialists are confident in the use of these drugs according to the age of patients (s.4.2). Maybe some specialists believe that age from birth certificate is not a pressing criterion, while physiological age and other aspects need to be considered.

Once the antidepressant drug has been chosen, treatment regimens should last 3 months at least, in the opinion of most specialists. It is reasonable considering that the National Institute for Health and Clinical Excellence (NICE) guidelines on depression advise a minimum of 6-month treatment [43].

Concerning the ideal features of the antidepressant therapy in AD, the panel focuses on the efficacy on anhedonia (s.4.5), that is believed as the main symptom of depression in AD, as already discussed, and on cognitive symptoms (s.4.6). According to these preconditions, most specialists identified SSRI and the multimodal antidepressant as a valid therapeutic choice for treating depression in AD (s.4.4). This result is in line with most guidelines, based largely on clinical experience [40].

In the opinion of specialists, vortioxetine, the new multimodal antidepressant, may have some potential benefits on anhedonia and on cognitive symptoms. Effectively, a previous study on a large population of patients with MDD showed significant short- and long-term efficacy against anhedonia [44, 45]. Regarding the procognitive effects of vortioxetine, they were thought to be related to the agent’s modulation of neurotransmitters, and less likely related purely to improvements in depressive symptoms [12]. On the other hand, a previous study showed that in patients with AD vortioxetine had a beneficial effect on cognitive performances, even if compared with other antidepressant drugs [17]. This data has been confirmed in a very recent analysis of a post-marketing surveillance [46]. Quite the opposite, a recent double-blind, placebo-controlled study in patients with AD showed no differences in terms of depressive symptoms, cognitive function, and impact on daily living activities in subjects treated with vortioxetine compared to placebo [18]. Nevertheless, even if few and conflicting data are actually available on the efficacy of vortioxetine in AD, considering the mechanism of action of this drug and previous data on its procognitive effect [47–49], vortioxetine seems to be a promising alternative in patients with AD and depression in the opinion of most panelists.

It is believed by most specialists that serotoninergic antidepressant could act with antidementia medication—specifically cholinesterase inhibitors (AChEIs)—on mood and cognition in a synergistic manner. A previous study confirmed that in AD patients treated with AChEIs, SSRIs may exert some degree of protection against the negative effects of depression on cognition [43]. The authors found that patients depressed and not treated with SSRIs worsened in terms of mean change of MMSE score compared with those depressed and treated with SSRI. Further, also cholinesterase inhibitors could have some effects on depression: donepezil has been found to be associated with improvement of depression in AD patients, which was measured mainly by screening tools for BPSD [50]. Also rivastigmine and galantamine have been proven to improve depressive symptoms in patients with AD [19, 20], even if with conflicting results [51]. On the other hand, in vitro studies demonstrated that SSRIs are able to interfere with the APP metabolism in vitro [52].

Finally, non-pharmacological therapies are considered. A mild consensus was reached on the effectiveness of cognitive-behavior therapy (s.4.11). Various international guidelines [41, 43] suggest that therapy for depression in dementia should include a variety of non-pharmacologic options, as cognitive behavioral therapy, reminiscence therapy, multi-sensory stimulation, animal-assisted therapy, exercise, and stimulation-oriented treatment, even though with a lower degree of evidence [11]. Probably all non-pharmacological therapies need to be more considered and an extensive knowledge and employment of these options is still lacking between specialists.

## Conclusion

The results of this Delphi questionnaire highlight some crucial aspects of depression in AD. Depression in older adults represents a risk factor for dementia and in some conditions is considered a prodromal condition; the consequence in clinical practice is that depressed people have to be carefully evaluated regarding cognitive disturbances and their development. Further, depression in the context of AD seems to have specific features in comparison with major depressive disorder. Nevertheless, the accurate background and assessment of depressive symptoms in AD still represents a challenge for the clinicians, because the complex overlap between affective and cognitive symptoms makes the cognitive assessment and the administration of specific scales for mood difficult.

The complex interaction between depression and dementia is probably due to a shared pathogenetic background that includes different neurotransmitter systems, firstly the cholinergic system. In consequence of the specificity of depressive symptoms in AD and of their difficult assessment, the clinicians do not refer to any diagnostic criteria. In fact, the DSM 5 diagnostic criteria for MDD are not able to detect the specific aspects of depression in presence of dementia. Concerning treatment, although evidence is mixed, antidepressant drugs are generally considered the main option for depression in dementia, according to previous guidelines. In order to limit side effects, SSRIs and the most recent multimodal antidepressant are preferred by clinicians. In particular, the procognitive effect of vortioxetine such as the effect on anhedonia seems to be appealing for the treatment of depression in AD.

In summary, this work integrates the opinions of a group of Italian specialists in a wide debate on depression in AD and contributes to this topic trying to explain the most controversial etiological and clinical aspects of depression and dementia. Furthermore, some clinical aspects, as the use of reliable diagnostic criteria, are still unmet and require future investigations and specific recommendations.
